# Supplementary material for: Identification of pathogenic Leptospira species and serovars in New Zealand using metabarcoding
Source: PLoS One. 2021 Sep 29;16(9):e0257971. doi: 10.1371/journal.pone.0257971 (PMC8480790; doi:10.1371/journal.pone.0257971)
Supplement: S1 Fig — Conventional PCR with glmU MLST_2 primers (top) and glmU_DW primers (bottom). LD = 1kb+ ladder, NC = negative control, PC = positive control (L. borgpeterseneii serovar Hardjo DNA), lanes 1–10 = PCR products amplified from gDNA extracted from cattle urine. Samples 6, 7, 9 and 10 from the bottom gel were verified as Leptospira with Sanger sequencing. (DOCX) [file pone.0257971.s001.docx]

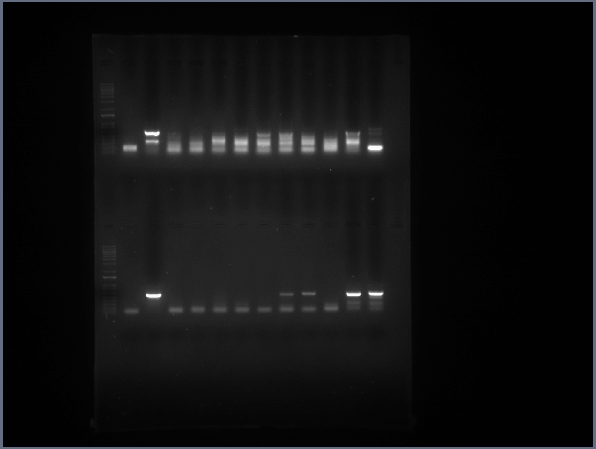
 **S1 Fig:** Conventional PCR with glmU MLST_2 primers (top) and glmU_DW primers (bottom). LD = 1kb+ ladder, NC = negative control, PC = positive control (*L. borgpeterseneii* serovar Hardjo DNA), lanes 1-10 = PCR products amplified from gDNA extracted from cattle urine. Samples 6, 7, 9 and 10 from the bottom gel were verified as *Leptospira* with Sanger sequencing.

LD NC PC 1 2 3 4 5 6 7 8 9 10

LD NC PC 1 2 3 4 5 6 7 8 9 10

LD NC PC 1 2 3 4 5 6 7 8 9 10
