# Supplementary material for: Identification of pathogenic Leptospira species and serovars in New Zealand using metabarcoding
Source: PLoS One. 2021 Sep 29;16(9):e0257971. doi: 10.1371/journal.pone.0257971 (PMC8480790; doi:10.1371/journal.pone.0257971)
Supplement: S1 Table — (DOCX) [file pone.0257971.s002.docx]

**Supplementary Table** 1: Bacterial strains used for metabarcoding controls

| **Genomopecies** | **Serogroup** | **Serovar** | **Strain** | **Country** | **Host** | **Year** | **Source** |
| --- | --- | --- | --- | --- | --- | --- | --- |
|  | Ballum | Ballum | Mus 127 | Denmark | Mouse | 1944 | Leptospirosis Reference Centre, Queensland |
|  | Serjoe | Balcanica | RL16 | New Zealand | Deer | 2017 | mEpiLab, New Zealand |
|  |  | Hardjo type Bovis | 205 | New Zealand | Sheep | 2009 | mEpiLab, New Zealand |
|  | Tarassovi | Tarassovi | Perepelitsin | Australia | Human | 1942 | Leptospirosis Reference Centre, Queensland |
| *Leptospira interrogans* | Icterohaemorrhagiae | Copenhageni | M20 | Denmark | Human | 1938 | Leptospirosis Reference Centre, Queensland |
|  | Pomona | Pomona | Pomona_str68 | New Zealand | Skunk | 1977 | USA |
