# Supplementary material for: Identification of pathogenic Leptospira species and serovars in New Zealand using metabarcoding
Source: PLoS One. 2021 Sep 29;16(9):e0257971. doi: 10.1371/journal.pone.0257971 (PMC8480790; doi:10.1371/journal.pone.0257971)
Supplement: S2 Table — (DOCX) [file pone.0257971.s003.docx]

**Supplementary Table 2:** Primers analysed or used in this study

| **LOCUS** | **Forward/**  **Reverse** | **Primer name** | **Primer sequence** | **Position** | **Conserved primer (Yes/No)** | **Degenerate primer**  **(Yes/No)** | **Degenerate positions** | **No. of mismatches**** | **Source** |
| --- | --- | --- | --- | --- | --- | --- | --- | --- | --- |
| **16S** | F | P16S-8UA | AGAGTTTGATCMTGGCTCAG | 4 | Y | Y | 1 | 0 | [1] |
|  | F | rrs_outer_F | CTCAGAACTAACGCTGGCGGCGCG | 19 | Y | N | 0 | 0 | [2] |
|  | F | Lep1 | GGCGGCGCGTCTTAAACATG | 34 | Y | N | 0 | 0 | [3] |
|  | F | Lepto16S11F | GGCGGCGCGTCTTAAACATGC | 34 | Y | N | 0 | 0 | [4] |
|  | F | 16S13 | CGGCGCGTCTTAAACATG | 35 | Y | N | 0 | 0 | [5] |
|  | F | rrs_inner_F | CTGGCGGCGCGTCTTA | 33 | Y | N | 0 | 0 | [2] |
|  | F | rrs_F1 | CATGCAAGTCAAGCGGAGTA | 50 | Y | N | 0 | 0 | MLST_3 |
|  | F | Lepat1 | GAGTCTGGGATAACTTT | 119 | Y | N | 0 | 0 | [5] |
|  | F | LeptoF^171^ | CCCGCGTCCGATTAG | 214 | N | N | 0 | v(0,1) | [6] |
|  | F | 16S_2_F | TAAAGGCTCACCAAGGCGAC | 242 | N | N | 0 | v(0,1) | [1] |
|  | F | 16S_1_F | GCGTAGGCGGACATGTAAGT | 546 | Y | N | 0 | 1 | [1] |
|  | F | LG1 | CGGTGAAATGCGTAGATATC | 656 | Y | N | 0 | 0 | [7] |
|  | R | LeptoR^258^ | TCCATTGTGGCCGR^A/G^ACAC | 301 | Y | Y | 1 | 0 | [6] |
|  | R | Lep2 | TTCCCCCCATTGAGCAAGATT | 364 | Y | N | 0 | 0 | [3] |
|  | R | Lepat2 | TCACATCGYTGCTTATTTT | 448 | N | Y | 1 | v(1) | [5] |
|  | R | 16S_2_R | TTAGCCGGTGCTTTAGGCAG | 477 | Y | N | 0 | 0 | [1] |
|  | R | 16S522 | TCCGCCTACACACCCTTTAC | 556 | Y | N | 0 | 1 | [5] |
|  | R | rrs_inner_R | GTTTTCACACCTGACTTACA | 578 | Y | N | 0 | 0 | [2] |
|  | R | rrs-R1 | AGTTGAGCCCGCAGTTTTC | 591 | Y | N | 0 | 0 | MLST_3 |
|  | R | 16S_1_R | AATCCCGTTCACTACCCACG | 756 | Y | N | 0 | 0 | [1] |
|  | R | rrs_outer_R | GGTTCGTTACTGAGGGTTAAAACCCCC | 828 | Y | N | 0 | 0 | [2] |
|  | R | LG2 | CGGTTTGTCACCGGCAGTTC | 1136 | Y | N | 0 | 0 | [7] |
|  | R | Lepto16S1338R | TGTGTACAAGGTCCGGGAAC | 1360 | Y | N | 0 | 0 | [4] |
|  | R | P16S_1485R | TACGGYTACCTTGTTACGACTT | 1476 | Y | Y | 1 | 0 | [1] |
| **LipL32** | F | LipL32_2_F | TGGCTATCTCCGTTGCACTC | 20 | N | N | 0 | v(0,2) | [1] |
|  | F | LipL32_F1 | ATCTCCGTTGCACTCTTTGC | 25 | N | N | 0 | v(0,1) | MLST_2, MLST_3 |
|  | F | LipL32-45F* | AAGCATTACCGCTTGTGGTG | 45 | Y | N | 0 | 0 | [8] |
|  | F | Fwd_LipL32 | TCCCAGGGACAAACGAAACCGT | 119 | N | N | 0 | v(0,2) | [9] |
|  | F | LipL32_F2 | GGATCTGTGATCAACTATTAC | 180 | Y | N | 0 | 0 | [10] |
|  | F | LipL32_F3 | TCGCTGAAATRGGWGTTCGT | 288 | N | N | 0 | v(0,1) | [11] |
|  | F | LipL32_1_F | GCCGTAATCGCTGAAATGGG | 262 | N | N | 0 | v(0,1) | [1] |
|  | F | LipL32_F5 | CTAAGTTCATACCGTGATTT | 269 | Y | Y | 2 | 0 | [12] |
|  | F | LipL32_270F | CGCTGAAATGGGAGTTCGTATGATT | 270 | Y | N | 0 | 0 | [13] |
|  | F | LipL32F | AGAGGTCTTTACAGAATTTCTTTCACTACCT | 619 | Y | N | 0 | 0 | [14] |
|  | R | Rev_LipL32 | TGTTTCCATCGGCTAAACCGT | 229 | N | N | 0 | v(1,2) | [9] |
|  | R | LipL32_2_R | CCCATTTCAGCGATTACGGC | 281 | N | N | 0 | v(0,1) | [1] |
|  | R | LipL32_R2 | CGAACTCCCATTTCAGCGATTAC | 287 | Y | N | 0 | 0 | [10] |
|  | R | LipL32_286R* | GAACTCCCATTTCAGCGATT | 286 | Y | N | 0 | 0 | [8] |
|  | R | LipL32_R3 | CGCCTGGYTCMCCGATT | 325 | N | Y | 2 | 0 | [11] |
|  | R | LipL32_1_R | CTTTGGCGATTTGGTCAGGC | 451 | N | N | 0 | v(0,1) | [1] |
|  | R | LipL32_R1 | ACCATCATCATCATCGTCCA | 498 | N | N | 0 | v(0,1) | MLST_2, MLST_3 |
|  | R | LipL32_692R | CCAACAGATGCAACGAAAGATCCTTT | 692 | Y | N | 0 | 0 | [13] |
|  | R | LipL32R | TGGGAAAAGCAGACCAACAGA | 705 | N | N | 0 | v(0,1) | [14] |
|  | R | LipL32_R5 | TCTGACGCGACTAAGTAAT | 703 | N | N | 0 | v(2,3) | [12] |
| **secY** | F | SecYII | GAATTTCTCTTTTGATCTTCG | 572 | N | N | 0 | v(0,1) | [15] |
|  | F | G2 | GGAAAACAAATGGTCGGAAG | 750 | Y | N | 0 | 0 | [7] |
|  | F | SecY-F1 | ATGCCGATCATTTTTGCTTC | 829 | N | N | 0 | v(0,1,2) | MLST_3 |
|  | F | SecY-IVF | GCGATTCAGTTTAATCCTGC | 1027 | N | N | 0 | v(0,2) | [16] |
|  | R | G1 | CTGAATCGCTGTATAAAAGT | 1035 | N | N | 0 | v(1,1) | [7] |
|  | R | SecY_IVR | GAGTTAGAGCTCAAATCTAAG | 1229 | N | N | 0 | v(0,2) | [15] |
|  | R | SecY_R1 | CCGTCCCTTAATTTTAGACTTCTTC | 1377 | N | N | 0 | v(0,1) | MLST_3 |
|  | R | SecY_R2 | CCTTCCTTTAATTTTAGACTTTTTC | 1377 | N | N | 0 | v(2,3) | MLST_3 |
| **flaB** | F | L_flaB_F1 | TCTCACCGTTCTCTAAAGTTCAAC | 34 | N | N | 0 | v(0,1) | [17] |
|  | F | FlaB_1_F | GCTCGTGCAGGTGGAAGTAT | 324 | N | N | 0 | v(0,1) | [1] |
|  | F | FlaB_2_F | GCTAACGACGTGATCGGTCT | 571 | N | N | 0 | V(0,4) | [1] |
|  | F | LflaB_QF2 | CTTACGARGATCATGAAGCAGAG | 604 | N | Y | 2 | 0 | [18] |
|  | R | FlaB_1_R | GCCTTTGAAGTCATCGTGCC | 515 | N | N | 0 | v(0,2) | [1] |
|  | R | FlaB_2_R | CGAGACAACTTCTTCCGCCA | 753 | N | N | 0 | 1 | [1] |
|  | R | LflaB_QR2 | TGTTTTGTGGTCAGCGAGACA | 767 | Y | N | 0 | 0 | [18] |
|  | R | L_flaB_R1 | CTGAATTCGGTTTCATATTTGCC | 826 | N | N | 0 | v(0,1) | [17] |
| **adk** | F | adk_F1 | GGGCTGGAAAAGGTACACAA | 32 | N | N | 0 | v(0,2) | MLST_2 |
|  | F | adk_F2 | ACATTATCTTCATGGGACCTCC | 8 | N | N | 0 | V(0,1,2,3) | MLST_3 |
|  | R | adk_R1 | ACGCAAGCTCCTTTTGAATC | 544 | N | N | 0 | v(0,2,3) | MLST_2 |
|  | R | adk_R2 | TTACACAAGCTCCCTTTGAAT | 546 | N | N | 0 | v(0,2) | MLST_3 |
| **glmU** | F | glmU_F* | AGGATAAGGTCGCTGTGGTA | 17 | Y | N | 0 | 0 | MLST_2 |
|  | F | glmU_DW_F* | CCCGTATGAAAACGGATCAGCC | 53 | Y | N | 0 | 0 | This study |
|  | F | GDW_long_F* | TTTCTGTTGGTGCTGATATTGCCCCGTATGAAAACGGATCAGCC | 53 | Y | N | 0 | 0 | This study |
|  | R | glmU_R* | AGTTTTTTTCCGGAGTTTCT | 634 | Y | N | 0 | 0 | MLST_2 |
|  | R | glmU_DW_R* | ATTCTCCCTGAGCGTTTTGATTTC | 581 | Y | N | 0 | 0 | This study |
|  | R | GDW_long_R* | ACTTGCCTGTCGCTCTATCTTCATTCTCCCTGAGCGTTTTGATTTC | 581 | Y | N | 0 | 0 | This study |
| **LigB** | F | PSBF | ACWRVHVHRGYWDCCTGGTCYTCTTC | 2125 | N | Y | 7 | 0 | [15] |
|  | F | LigB_LP1 | TCGTTTTAGAATCGATAG | 4797 | Y | N | 0 | 0 | [19] |
|  | R | PSBR | TARRHDGCYBTAATATYCGRWYYTCCTAA | 2513 | N | Y |  | 0 | [15] |
|  | R | LigB_LP2 | ATACTTCCATTATGTA | 5048 | Y | N | 0 | 0 | [19] |
| **LipL41** | F | LipL41_F1 | TAGGAAATTGCGCAGCTACA | 50 | Y | N | 0 | 0 | MLST_2,  MLST_3 |
|  | F | LipL41_1_F | GTGCAGACGCAATCAACGAA | 293 | N | N | 0 | v(0,3) | [1] |
|  | F | LipL41_2_F | CGTAGGTTTGGCTGTTGAAGC | 147 | N | N | 0 | v(0,1,2,3) | [1] |
|  | R | LipL41_1_R | GCGAAACCTGCCACTTTCAA | 476 | N | N | 0 | v(0,1) | [1] |
|  | R | LipL41_2_R | GCGTCTGCACGTTTACTCAG | 283 | N | N | 0 | V(0,2,4) | [1] |
|  | R | LipL41_R1 | GCATCGAGAGGAATTAACATCA | 569 | N | N | 0 | v(0,2,3) | MLST_2,  MLST_3 |
| **LipL31** | F | LipL31_F | TCGATGCGATGAGTCGAGTT | 143 | N | N | 0 | v(1,2) | [1] |
|  | R | LipL31_R | AACCGTCTTTTTCAGCTGCG | 322 | N | N | 0 | v(0,3) | [1] |
| **icdA** | F | icdA_F1 | GGGACGAGATGACCAGGAT | 44 | Y | N | 0 | 0 | MLST_2, MLST_3 |
|  | R | icdA_R1 | CTTTTTTGAGATCCGCAGCTTT | 718 | N | N | 0 | v(0,3) | MLST_2, MLST_3 |
| **gyrB** | F | 2For* | TGAGCCAAGAAGAAACAAGCTACA | 2 | N | N | 0 | v(0,1,2,2) | [20] |
|  | R | 504Rev* | MATGGTTCCRCTTTCCGAAGA | 504 | N | Y | 2 | v(2,3) | [20] |
| **mreA** | F | mreA_F1 | GGCTCGCTCTYGACGGAAA | 713 | N | Y | 2 | v(0,1) | MLST_2 |
|  | R | mreA_R1 | TCCRTAACTCATAAAMGACAAAGG | 1431 | N | Y | 2 | 0 | MLST_2 |
| **pntA** | F | pntA_F1 | TAGGAAARATGAAACCRGGAAC | 239 | N | Y | 2 | v(0,2) | MLST_2 |
|  | R | pntA_R1 | AAGAAGCAAGATCCACAAYTAC | 859 | N | Y | 1 | v(0,1) | MLST_2 |
| **rpoB** | F | Lept_1900f | CCTCATGGGTTCCAACATGCA | 1881 | Y | N | 0 | 0 | [15] |
|  | R | Lept_2500R | CGCATCCTCRAAGTTGTAWCCTT | 2472 | N | Y | 2 | v(0,1) | [15] |

*Primers used in this study

**Zero represents a primer that is a perfect match *i.e.* zero mismatches against all lineages tested, v(1-4) represent the variable number of mismatches between the different lineages *e.g.* v(0,1) indicates some species have a perfect match while others have single nucleotide mismatch in the primer region.

1. Backstedt, B.T., et al., *Efficient Detection of Pathogenic Leptospires Using 16S Ribosomal RNA.* PLOS ONE, 2015. **10**(6): p. e0128913.

2. Boonsilp, S., et al., *Molecular detection and speciation of pathogenic Leptospiraspp. in blood from patients with culture-negative leptospirosis.* BMC Infectious Diseases, 2011. **11**(1): p. 338.

3. Mérien, F., et al., *Polymerase chain reaction for detection of Leptospira spp. in clinical samples.* Journal of Clinical Microbiology, 1992. **30**(9): p. 2219-2224.

4. Ganoza, C.A., et al., *Determining risk for severe leptospirosis by molecular analysis of environmental surface waters for pathogenic Leptospira*. 2006, PUBLIC LIBRARY SCIENCE.

5. Mason, M.R., et al., *Distribution and Diversity of Pathogenic Leptospira Species in Peri-domestic Surface Waters from South Central Chile.* PLOS Neglected Tropical Diseases, 2016. **10**(8): p. e0004895.

6. Smythe, L.D., et al., *A quantitative PCR (TaqMan) assay for pathogenic Leptospira spp.* BMC Infect Dis, 2002. **2**.

7. Benacer, D., et al., *Determination of Leptospira borgpetersenii serovar Javanica and Leptospira interrogans serovar Bataviae as the persistent Leptospira serovars circulating in the urban rat populations in Peninsular Malaysia.* Parasites & Vectors, 2016. **9**(1): p. 117.

8. Stoddard, R.A., et al., *Detection of pathogenic Leptospira spp. through TaqMan polymerase chain reaction targeting the LipL32 gene.* Diagnostic Microbiology and Infectious Disease, 2009. **64**(3): p. 247-255.

9. Gentilini, F., et al., *A comparison of two real-time polymerase chain reaction assays using hybridization probes targeting either 16S ribosomal RNA or a subsurface lipoprotein gene for detecting leptospires in canine urine.* Journal of Veterinary Diagnostic Investigation, 2015. **27**(6): p. 696-703.

10. Mullan, S. and T.H. Panwala, *Polymerase Chain Reaction: An Important Tool for Early Diagnosis of Leptospirosis Cases.* Journal of Clinical and Diagnostic Research : JCDR, 2016. **10**(12): p. DC08-DC11.

11. Rojas, P., et al., *Detection and quantification of leptospires in urine of dogs: a maintenance host for the zoonotic disease leptospirosis.* European Journal of Clinical Microbiology & Infectious Diseases, 2010. **29**(10): p. 1305-1309.

12. Gökmen, T.G., et al., *Comparison of 16s rRNA-PCR-RFLP, LipL32-PCR and OmpL1-PCR methods in the diagnosis of leptospirosis* Revista do Instituto de Medicina Tropical de São Paulo, 2016. **58**.

13. Vein, J., et al., *Adaptation of a real-time PCR method for the detection and quantification of pathogenic leptospires in environmental water.* Canadian Journal of Microbiology, 2012. **58**(7): p. 828-835.

14. Villumsen, S., et al., *Novel TaqMan® PCR for detection of Leptospira species in urine and blood: Pit-falls of in silico validation.* Journal of Microbiological Methods, 2012. **91**(1): p. 184-190.

15. Cerqueira, G.M., et al., *Bioinformatics Describes Novel Loci for High Resolution Discrimination of Leptospira Isolates.* PLOS ONE, 2010. **5**(10): p. e15335.

16. Ahmed, A., et al., *Development and Validation of a Real-Time PCR for Detection of Pathogenic Leptospira Species in Clinical Materials.* PLOS ONE, 2009. **4**(9): p. e7093.

17. Kawabata, H., et al., *flaB-Polymerase Chain Reaction (flaB-PCR) and Its Restriction Fragment Length Polymorphism (RFLP) Analysis Are an Efficient Tool for Detection and Identification of Leptospira Spp.* Microbiology and Immunology, 2001. **45**(6): p. 491-496.

18. Iwasaki, H., et al., *Combined antibody and DNA detection for early diagnosis of leptospirosis after a disaster.* Diagnostic Microbiology and Infectious Disease, 2016. **84**(4): p. 287-291.

19. Benacer, D., et al., *A duplex endpoint PCR assay for rapid detection and differentiation of Leptospira strains.* Revista da Sociedade Brasileira de Medicina Tropical, 2017. **50**: p. 239-242.

20. Slack, A.T., et al., *Identification of pathogenic Leptospira species by conventional or real-time PCR and sequencing of the DNA gyrase subunit B encoding gene.* BMC Microbiology, 2006. **6**: p. 95-95.
